# The effect of acute ventilation-perfusion mismatch on respiratory heat exchange in a porcine model

**DOI:** 10.1371/journal.pone.0254399

**Published:** 2021-07-12

**Authors:** Maximilian Edlinger-Stanger, Martin-Hermann Bernardi, Katharina Kovacs, Michael Mascha, Thomas Neugebauer, Stefan Böhme, Nathan Ayoubi, Nico Christofi, James Garry, Neal Fleming, Michael Hiesmayr

**Affiliations:** 1 Department of Cardiothoracic Anaesthesia, Intensive Care Medicine, Medical University of Vienna, Vienna, Austria; 2 Department of Anaesthesia, Intensive Care Medicine and Pain Medicine, Medical University of Vienna, Vienna, Austria; 3 Rostrum Medical Innovations Inc., Vancouver, Canada; 4 University of California Davis, Davis, California, United States of America; Tokyo Joshi Ika Daigaku Toyo Igaku Kenkyujo Clinic, JAPAN

## Abstract

**Background:**

Respiratory heat exchange is an important physiological process occurring in the upper and lower respiratory tract and is usually completed when inspired gases reach the alveoli. Animal and human studies demonstrated that heat exchange can be modulated by altering pulmonary ventilation and perfusion. The purpose of this study was to examine the effect of acute ventilation-perfusion (V/Q) mismatch on respiratory heat exchange. In clinical practice, monitoring respiratory heat exchange might offer the possibility of real-time tracking of acute V/Q-mismatch.

**Methods:**

In 11 anesthetized, mechanically ventilated pigs, V/Q-mismatch was established by means of four interventions: single lung ventilation, high cardiac output, occlusion of the left pulmonary artery and repeated whole-lung lavage. V/Q-distributions were determined by the multiple inert gas elimination technique (MIGET). Respiratory heat exchange was measured as respiratory enthalpy using the novel, pre-commercial VQm^™^ monitor (development stage, Rostrum Medical Innovations, Vancouver, CA). According to MIGET, shunt perfusion of low V/Q compartments increased during single lung ventilation, high cardiac output and whole-lung lavage, whereas dead space and ventilation of high V/Q compartments increased during occlusion of the left pulmonary artery and whole-lung lavage.

**Results:**

Bohr dead space increased after pulmonary artery occlusion and whole-lung lavage, venous admixture increased during single lung ventilation and whole-lung lavage, P_a_O_2_/F_i_O_2_ was decreased during all interventions. MIGET confirmed acute V/Q-mismatch. Respiratory enthalpy did not change significantly despite significant acute V/Q-mismatch.

**Conclusion:**

Clinically relevant V/Q-mismatch does not impair respiratory heat exchange in the absence of additional thermal stressors and may not have clinical utility in the detection of acute changes.

## Introduction

Heat and water exchange are important processes occurring in the respiratory tract and are proposed to be dependent on matching of ventilation and perfusion. Under physiological conditions, this transfer of heat and water is usually completed before inspired gases reach the alveoli. Both the upper and lower respiratory tract participate in thermal conditioning and their relative contribution depends on the thermal burden imposed on the respiratory system. Tidal volumes, minute ventilation and properties of the inhaled air itself influence thermal conditioning. During quiet breathing, conditioning is completed within the upper respiratory tract, whereas during high-flow states (exercise, hyperventilation) or during “thermal challenges” (such as breathing dry, cold air), the lower respiratory tract contributes to thermal conditioning, probably until the 10-15^th^ generation of bronchioles [1–5]. Thermal resistance is generally lower in the distal airways compared to proximal airways owing to the smaller wall thickness [6–8]. Thus, pulmonary perfusion potentially modulates heat transfer in conditions where distal airways contribute to thermal conditioning. Experimental animal and human studies demonstrated the significance of pulmonary blood flow in thermal conditioning showing that pulmonary arterial blood flow is the major source of heat in the lower respiratory tract [9]. There is a direct correlation between pulmonary blood flow (and hence cardiac output) on “thermal conductance” of the respiratory system [10]. Temperature differences between pulmonary blood and expired gases are diminished significantly with increasing cardiac output. Furthermore, the kinetics of heat exchange are modulated by cardiac output. For example, the specific time-constant of temperature decay after switching from warm, humid air to dry, cold air is increased when cardiac output falls [6,9,11]. Bronchial blood flow, on the other hand, is only 1/50-1/100 of pulmonary blood flow and does not significantly affect expired gas temperature in isolated lungs when normal pulmonary perfusion is preserved. Rather, bronchial blood flow from systemic bronchial arteries mainly regulates water exchange across the bronchial mucosa [6,10–14]. Thus, both ventilatory (tidal volume, velocity of gas flow, minute ventilation, physical properties of air) and perfusion-related (cardiac output, intrathoracic blood volume, bronchial vs. pulmonary blood flow) variables influence the transfer of heat in the respiratory system.

Under physiological conditions in the resting state, thermal conditioning generally is completed before gases reach the alveoli. Most of this conditioning occurs in the nasal cavity and pharynx [2,8,15]. However, under stressed conditions such as hyperventilation, exercise, breathing of cold dry air and after bypassing the nasal cavity and pharynx by means of endotracheal intubation, the significance of the pulmonary and bronchial circulation in terms of heat exchange becomes evident [4,6,9–11,13]. Thus, matching of ventilation and perfusion could be an important variable during heat transfer [6].

We hypothesized that acute ventilation-perfusion (V/Q) mismatch serves as an alternative stressor that limits the efficiency of thermal conditioning of inspired air. The goal of this study was to investigate the specific effects of acute V/Q mismatch on pulmonary heat exchange. The relationship between V/Q mismatch and heat exchange is of potential clinical relevance, as the minimally invasive measurement of pulmonary heat exchange via the endotracheal tube might provide a monitoring tool for V/Q mismatch at the bedside.

To test this hypothesis, we designed five clinically relevant pathophysiological interventions mimicking different etiologies of acute V/Q mismatch or common clinical interventions: Single-lung ventilation, high cardiac output, wedging of the left main pulmonary artery, repeated whole-lung lavage and a PEEP (Positive End-Expiratory Pressure) trial after whole-lung lavage. Respiratory enthalpy was measured using the novel VQm^™^ pre-commercial monitor (Rostrum Medical Innovations, Vancouver, CA). In daily clinical practice, assessment of V/Q distributions using multiple inert gas elimination technique (MIGET), the current gold standard, cannot be employed routinely due to high technical and personnel requirements. Therefore, alternative techniques for evaluation of V/Q matching at the bedside, such as respiratory enthalpy, are of great interest.

## Materials and methods

### Ethics approval

The experimental protocol was approved by the Austrian Federal Ministry of Education, Science and Research (GZ.:66.009/0176-WF/V/3b/2016) and experiments were carried out according to national animal research regulations (Directive 2010/63/EU, Tierversuchsverordnung 2012).

### Anesthesia and preparation

Animals were housed for seven days before the study under controlled, standardized conditions (day:night rhythm 12:12 hours, room temperature 22±2°C, humidity 55±10%), kept in groups and had free access to water. All animals were sedated on the morning of the experiment and kept under general anesthesia as described below in order to ensure wellbeing, amnesia, hypnosis and analgesia.

11 pigs (1^st^ series #1–6: 71±11kg, 2^nd^ series #7–11: 56±4kg; Österreichische Edelschweine, scientific research animal breeder in Böheimkirchen, Lower Austria, Austria) were premedicated with ketamine (0.25mg/kg) and midazolam (0.15mg/kg). For administration of induction agents, a peripheral ear vein was cannulated. General anesthesia was induced and maintained with propofol (1200mg/h), fentanyl (500μg/h) and rocuronium-bromide (50mg/h). After intubation, initial ventilator settings (volume-controlled) were: TV 10ml/kg, F_i_O_2_ 0.3, PEEP 3 cmH_2_O, respiratory rate (RR) adjusted to achieve end-tidal CO_2_ (etCO_2_) of 35-45mmHg. The respirator delivered fresh, dry, unconditioned gas. A bronchus blocker (EZ-blocker^™^, Teleflex Medical Europe^®^, Athlone, Ireland) was positioned under bronchoscopic guidance. A central venous catheter (CVC) (Teleflex Medical Europe^®^, Athlone, Ireland) was inserted via the right or left external jugular vein and additional arterial cannulation of the right or left common carotid artery was performed for continuous blood pressure measurement and blood sampling. A pulmonary arterial catheter (PAC) (7.0 French, Swan-Ganz thermodilution catheter, Baxter Inc., Deerfield, Illinois, USA) was introduced via the right external jugular vein. A balloon valvuloplasty catheter (Tyshak II^®^, NuMED Inc., Hopkinton, NY, USA) was advanced into the left main pulmonary artery (LPA) under fluoroscopy in the following fashion: 1. placement of second PAC in the LPA via the right femoral vein 2. introduction of 0.025-inch guidewire via this PAC, 3. removal of PAC and introduction of a pig-tail catheter, 4. introduction of a 0.035-inch guidewire into LPA, 5. advancement of the balloon valvuloplasty catheter into LPA. The balloon was inflated under fluoroscopy using diluted contrast medium for final positioning. We chose the most proximal position that was still hemodynamically tolerated without the use of excessive doses of vasopressin. Suprapubic catheterization for urinary output monitoring was performed. Body temperature was kept between 38–40°C. For hemodynamic stabilization, animals received a continuous infusion of Ringer’s lactate (up to 10ml/kg/h). Whenever rapid hemodynamic instability occurred, additional doses of phenylephrine or adrenaline were administered. Humane endpoints were: persistent malignant arrhythmia (ventricular tachycardia or fibrillation, asystole, pulseless electrical activity), cardiac arrest. At the end of the experiments, all animals were euthanized using potassium chloride under deep general anesthesia. One animal deceased prematurely due to a massive air embolism during the preparation phase.

### Micropore membrane inlet mass spectrometry multiple inert gas elimination technique (MMIMS-MIGET)

In order to determine the effect and extent of our pathophysiological interventions we assessed V/Q distributions by means of micropore membrane inlet mass spectrometry MIGET (MMIMS-MIGET) [16]. MMIMS-MIGET combines mass spectrometry with MIGET and allows for faster analysis of V/Q distributions [17].

The MMIMS-MIGET technique has been described in detail previously [16,18–21]. Six inert gases (Sulphur hexafluoride, Krypton, Desflurane, Enflurane, Diethyl ether and Acetone) were dissolved in normal saline and infused continuously via the CVC after meticulous de-airing. Roller pumps were used to provide a stable flow rate of 1000ml/h and 3000ml/h in-between interventions and during interventions, respectively.

Samples (5ml) were taken simultaneously from systemic arterial and mixed venous blood over 5–10 breaths using air-tight EDTA-primed glass syringes. Samples were kept at 37°C during interventions and processed immediately thereafter. Inert gas retention and V/Q distributions were measured using MMIMS-MIGET (Oscillogy LLC, Folsom, USA). Dead space values were not calculated by MMIMS-MIGET in this study. The Enghoff-Bohr equation sufficiently detected trends in dead space ventilation upon acute V/Q shifts. Furthermore, good agreement between MMIMS-MIGET derived dead space and the Bohr equation has been demonstrated [22].

### Measurement of respiratory enthalpy

A pre-commercial apparatus (VQm^™^ monitor, Rostrum Medical Innovations, Vancouver, CA) was designed that allowed the total enthalpy present in an air flow to be measured. This system consisted of two tubular devices, hereafter referred to as ‘cores’, through which air could pass. A one-way valve (Hans-Rudolph 1810 series) was present in the airway circuit to ensure that air flowed through the cores such that one core only experienced inhaled air, and the other core carried both inhaled and exhaled airflows.

A perforated metal membrane was positioned in each core such that motion of air through the core would cause a slight but repeatable pressure drop between the core’s inlet and its exhaust port. Two hoses allowed the absolute pressure to be measured on either side of this membrane, allowing the volumetric flow rate of the air to be determined. Each core also contained a small chamber, from which air could be drawn by a distant sampling pump. This chamber was connected to the airway through a narrow opening of 1mm diameter. Inside this chamber a fine gauge ‘T’ type thermocouple (Omega CO-001) and a fast-acting hygrometer (Honeywell HIH-4000) were positioned so that air drawn by a distant sampling pump would be pulled from the main air flow path, through the narrow opening, and over the two sensors.

The thermocouple had a T90 response time of 3ms, and thus could readily record the changing temperature during an exhalation. By contrast, the hygrometer had a slower response time and was unable to resolve intra-breath variations in the humidity. However, a record of the average enthalpy content in an exhale could be made by operating the sampling pump briefly at the same point during a breath, for multiple consecutive breaths. In this way the small adjunct chamber would be filled with gas that progressively became closer in make up to that of the average exhalation. The calculation of the total heat energy in the air seen by both cores was computed by a separate monitor that housed a microcontroller, the sampling pump, pressure transducers and associated equipment. Extensive tests with a dedicated calibration system that was able to produce air streams with controlled temperatures and relative humidity levels had shown that the sensors were able to report the enthalpy of an airstream with an accuracy of approximately ±5%, depending on the flow speed of the air through the device.

After a planned interim analysis following the first six experiments, technical adaptations were made in order to deliver cooled inspired air. We were, however, unable to achieve significant cooling. Therefore, results from series 1 (animals #1–6) and series 2 (animals #7–11) were analyzed separately. Respiratory enthalpy is presented as mean enthalpy difference between inspiration and expiration.

### Calculation of venous admixture and dead space

Venous admixture was calculated according to the shunt equation with a porcine-specific routine [21,23,24]:

$$\frac{Q_{s}}{Q_{t}}=\frac{\left(C_{c}O_{2}-C_{a}O_{2}\right)}{\left(C_{c}O_{2}-C_{v}O_{2}\right)}$$

where Q_s_ = pulmonary shunt (L/min), Q_t_ = cardiac output (L/min), C_*c*_O_2_ = pulmonary capillary oxygen content, C_*a*_O_2_ = systemic arterial oxygen content and C_*v*_O_2_ = mixed venous oxygen content.

Dead space was calculated according to the Bohr-Enghoff equation [23]:

$$\frac{V_{Dphys}}{V_{T}}=\frac{\left(P_{a}{CO}_{2}-P_{et}{CO}_{2}\right)}{P_{a}{CO}_{2}}$$

where V_D*phys*_ = physiological dead space ventilation (L/min), V_T_ = total minute ventilation (L/min) (P_a_CO_2_ = arterial partial pressure of CO_2_, P_et_CO_2_ = end tidal partial pressure of CO_2_.

### Experimental protocol

After induction of anesthesia and the preparatory phase a stabilization period of 5min was allowed at baseline ventilator settings. Recruitment maneuvers were performed prior to baseline measurements and before establishing interventions. 15min after establishing a new intervention, hemodynamic data were recorded, and blood sampled as triplicates in 5min intervals. We performed the following pathophysiological interventions:

- Single lung ventilation (SLV) in order to produce atelectasis, increased venous admixture and low V/Q regions [19,25]: ventilation to the left side of the lung was blocked by inflating the EZ-blocker’s balloon located in the left main bronchus. Correct positioning was verified with bronchoscopy.
- High cardiac output (hCO) as may be present in sepsis in order to increase venous admixture [26,27]: in animals #1–6, high cardiac output was induced by continuous infusion of dobutamine. The infusion was titrated to achieve a heart rate of 130% of baseline.
- Wedging of the left main pulmonary artery (wLPA) as a reversible, closed-chest model of acute, massive pulmonary embolism in order to increase dead space ventilation [28]: during the preparatory phase, a balloon valvuloplasty catheter (Tyshak II^®^, 20x60mm, NuMED Inc., Hopkinton, NY, USA) was placed in the LPA. The balloon was inflated with diluted contrast media under fluoroscopy and placed in the most proximal location that was still hemodynamically tolerated. A significant drop in etCO_2_ and increased pulmonary artery pressures were observed upon inflation.
- Repeated whole-lung lavage (WLL) as an experimental model of acute respiratory distress syndrome (ARDS) [29,30]: whole-lung lavage was performed with 35ml/kg normal saline. Lavage was repeated until PaO2 was <70mmHg at FiO2 1.0 or a maximum of five lavages. PEEP was set at 0 cmH_2_O and F_i_O_2_ at 1.0 during this intervention.
- Positive end expiratory pressure trial (PEEPt): In animals #7–11, after the repeated whole-lung lavage intervention, PEEP was increased to 7 cmH_2_O for 10min, then 15 cmH_2_O for 10min, then 7 cmH_2_O for 10min and finally 0 cmH_2_O for 10min. At each PEEP level, respiratory enthalpy and hemodynamic variables were recorded. No MIGET samples were drawn during the PEEP trial.

Ventilator settings for baseline and interventions are given in Table 1.

**Table 1:** Ventilator settings.

Intervention	F_i_O_2_	PEEP (cmH_2_O)	TV (ml/kg)	RR (adjusted to)
Baseline	0.3*	3	10–12	etCO_2_ 35-45mmHg
SLV	0.3*	3	10–12	etCO_2_ < 45mmHg
hCO	0.3*	3	10–12	etCO_2_ 35-45mmHg
wLPA	0.3*	3	10–12	etCO_2_ 25-40mmHg
WLL	1.0	0	10–12	etCO_2_ 35-45mmHg
PEEP trial	1.0	7-15-7-0	10–12	etCO_2_ 35-45mmHg
Recruitment	0.3*	10	Variable; peak pressure 40cmH2O	20 cycles, I:E = 2:2sec

F_i_O_2_ fraction of inspired oxygen; PEEP, positive end expiratory pressure; TV, tidal volume; RR, respiratory rate; etCO_2_, end tidal CO_2_; wLPA, wedging of left main pulmonary artery; SLV, single lung ventilation; hCO, high cardiac output; WLL, whole-lung lavage*, adjusted to achieve an SpO2 of >90%.

**Fig 1:**
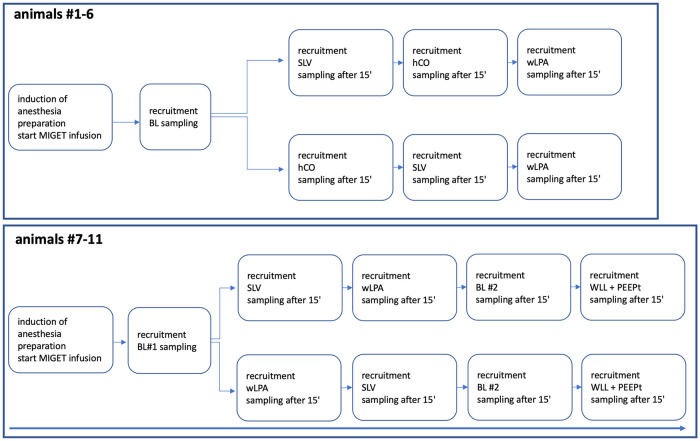
Experimental workflow. BL = baseline; SLV = single lung ventilation; hCO = high cardiac output; PEEPt = PEEP trial; wLPA = wedged left main pulmonary artery; WLL = whole-lung lavage; MIGET: Multiple inert gas elimination technique. The sequence of interventions was:
animals #1,2,5: BL–SLV–hCO–wLPAanimals #3, 4, 6: BL–hCO–SLV–wLPAanimal #7, 9, 11: BL #1—SLV–wLPA–BL #2 –WLL-PEEPtanimal #8, 10: BL #1—wLPA–SLV–BL #2 –WLL-PEEPt.

**Fig 2:**
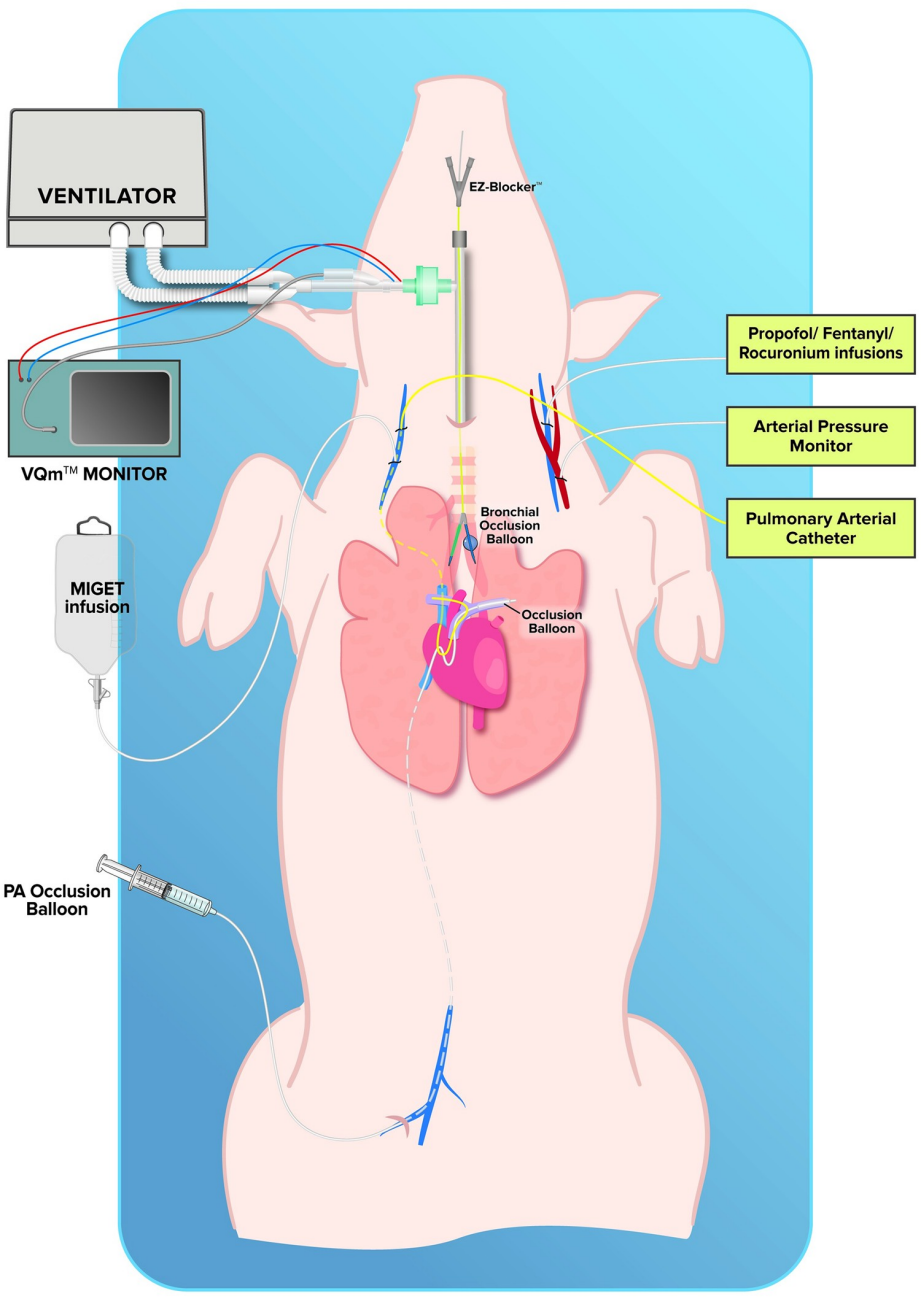
Experimental setup. MIGET = multiple inert gas elimination technique; PA = pulmonary artery.

Because not every animal was subjected to every intervention, the experimental workflow for animals #1–6 and #7–11 is shown in Fig 1. The experimental setup is depicted in Fig 2.

### Randomization

Single-lung ventilation, high cardiac output and wedging of the pulmonary artery were performed in random order. A sealed envelope system was used for randomization. Repeated whole-lung lavage had to be performed last due to the irreversible nature of the experimental ARDS model.

### Sample size estimation

A sample size of 14 animals was calculated based on the assumption that paired *t*-tests will be performed in order to detect a difference in means of one standard deviation for V/Q distributions with 90% power at alpha = 0.05. In case of dropouts, 11 animals would yield 80% power (STATA12.1).

### Statistical methods

The D’Agostino & Pearson normality test was used to test for Gaussian distribution of our data. Hemodynamic and respiratory parameters and results for respiratory enthalpy, dead space, Venous admixture and the P_a_O_2_/F_i_O_2_ ratio during interventions A-D were compared to baseline measurements using repeated-measures one-way ANOVA with Dunnett’s method (vs. baseline) for multiple testing. A *P* value < 0.05 was considered significant. Microsoft Excel and GraphPad Prism 7 (GraphPad Software, Inc., CA, USA) were used for data entry and statistical analysis.

## Results

### Hemodynamic parameters

With respect to baseline, significant differences were observed for PAP (increased during single-lung ventilation, wedged left main PA and repeated whole-lung lavage), CVP (increased during single-lung ventilation, wedged left main pulmonary artery and repeated whole-lung lavage), cardiac output (increased during high cardiac output), PIP (increased during single-lung ventilation and repeated whole-lung lavage) and etCO_2_ (decreased during wedged left main pulmonary artery) S_v_O_2_ (increased during high cardiac output and decreased during repeated whole-lung lavage) and SpO_2_ (decreased during repeated whole-lung lavage). Hemodynamic parameters are shown in Table 2.

**Table 2:** Hemodynamic parameters.

Intervention	HR (bpm)	SAP (mmHg)	PAP (mmHg)	CVP (mmHg)	SpO_2_ (%)	S_v_O_2_ (%)	CO (L/min)	T (°C)
Baseline	88±12	80±10	25±3	13±1	100	55±11	8.2±1.4	37.9±0.9
SLV	96±12	74±7	32±3*	15±1*	98±3	51±11	8.6±1.3	37.8±0.9
hCO	125±7*	80±7	29±3	14±1	100	65±8*	16±1.9	38.4±0.85
wLPA	99±15	77±10	40±10*	17±2*	95±7	52±11	8.4±1.9	37.8±1.1
WLL	104±11	76±6	43±5*	19±2*	87±13*	48±9*	7.2±0.27	36.9±0.9

All data are given as mean ± SD.

* indicates a significant difference with respect to baseline.

SAP, mean systemic arterial pressure; PAP, mean pulmonary arterial pressure; CVP, central venous pressure; SpO_2_, peripheral capillary oxygen saturation; S_v_O_2_, mixed venous oxygen saturation; CO, cardiac output; T, blood temperature, wLPA, wedging of left main pulmonary artery; SLV, single lung ventilation; hCO, high cardiac output; WLL, whole-lung lavage.

### Respiratory enthalpy, dead space, venous admixture and P_a_O_2_/F_i_O_2_

Respiratory enthalpy (reported as mean respiratory enthalpy difference) did not change significantly during the four pathophysiological interventions. Dead space increased after wedging of the left main pulmonary artery (*P < 0*.*01*) and lung lavage (*P < 0*.*01*), venous admixture increased during single-lung ventilation (*P < 0*.*01*) and repeated whole-lung lavage (*P < 0*.*01*) and P_a_O_2_/F_i_O_2_ decreased during all interventions (*P < 0*.*01*). Large SDs in animals #1–6 were due to low respiratory enthalpy in animals #5 and #6. V/Q distributions and hemodynamic parameters were comparable to animals #1–4.

**Fig 3:**
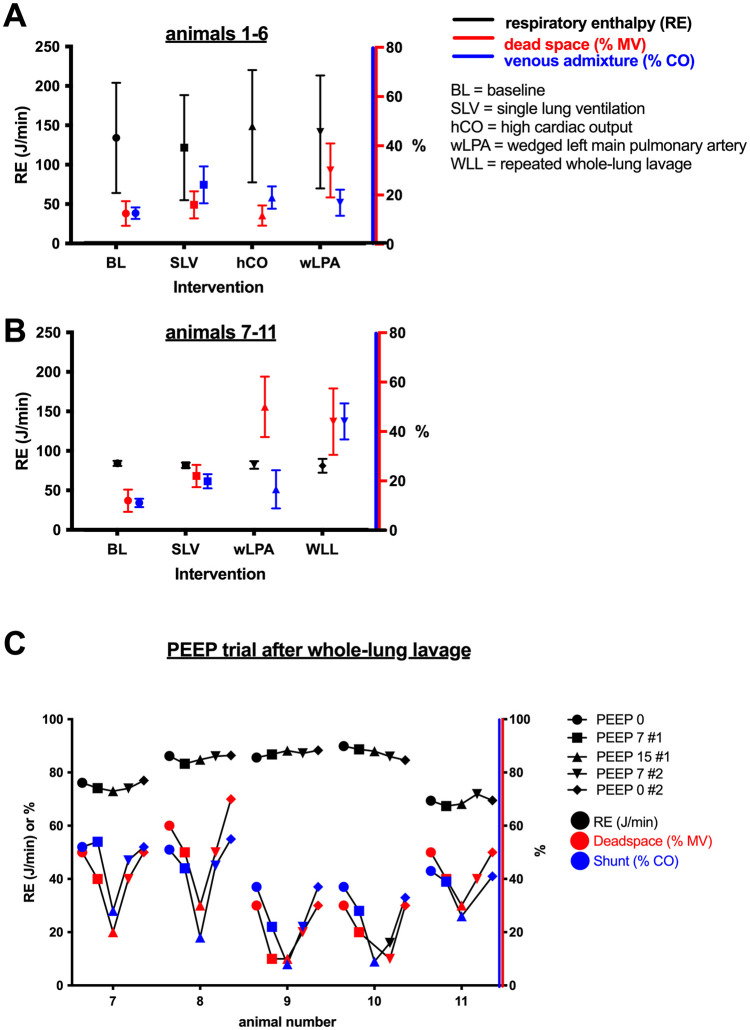
Results for respiratory enthalpy, dead space and venous admixture. A, pooled results for animal 1–6; B, pooled results for animals 7–11; C, results for each individual animal of series 7–11. For *A* and *B*, data are given as mean ±SD, for *C* data points are single measurements.

During the PEEP trial, both venous admixture and dead space were reduced with increasing PEEP levels (statistically significant for 15 PEEP when compared to zero PEEP). In contrast, effects on respiratory enthalpy were not significant. Table 3 and Fig 3 show results for respiratory enthalpy, dead space, venous admixture and P_a_O_2_/F_i_O_2_.

**Table 3:** Results for RE, PaO_2_/F_i_O_2_ ratio, dead space and venous admixture.

Intervention	RE (Joules/min)		PaO_2_/F_i_O_2_	Dead space (%)	Venous admixture (%)	MIGET shunt (%)
	*animals 1–6*	*animals 7–11*	*animals 1–11*	*animals 1–11*	*animals 1–11*	*animals 1–11*
Baseline	134.1 ± 69.96	84.2 ± 3.2	555 ± 93	12 ± 4	11 ± 2	5 ± 5
SLV	121.7 ± 66.78	81.8 ± 3.3	348 ± 7 *	19 ± 6	22 ± 6 *	19 ± 8
hCO	146.3 ± 70.50	*nm*	404 ± 33 *	12 ± 4	18 ± 5	5 ± 5
wLPA	141.6 ± 71.72	82.2 ± 4.8	350 ± 72 *	39 ± 15 *	16 ± 6	3 ± 3
WLL	*nm*	81.2 ± 8.8	85 ± 19 *	44 ± 13 *	44 ± 3 *	41 ± 3
PEEP 7 #1	*nm*	80.1 ± 9	125 ± 70	32 ± 15	37 ± 11	*nm*
PEEP 15	*nm*	80.4 ± 9.2	340 ± 131 **	18 ± 12	18 ± 8 **	*nm*
PEEP 7 #2	*nm*	81.0 ± 7.4	167 ± 93	32 ± 15	33 ± 14	*nm*
PEEP 0	*nm*	81.2 ± 7.8	71 ± 13	46 ± 15	44 ± 9	*nm*

All data are given as mean ± SD.

* indicates a significant difference with respect to baseline.

** indicates a significant difference with respect to the intervention WLL.

PEEP trial was only performed in animals 7–11.

RE, respiratory enthalpy; PaO_2_/F_i_O_2_ ratio of partial pressure of oxygen in arterial blood to fraction of inspired oxygen; LPA, left main pulmonary artery; PEEP, positive end expiratory pressure; nm, no measurement; wLPA, wedging of left main pulmonary artery; SLV, single lung ventilation; hCO, high cardiac output; WLL, whole-lung lavage.

### Results of MMIMS-MIGET

**Fig 4:**
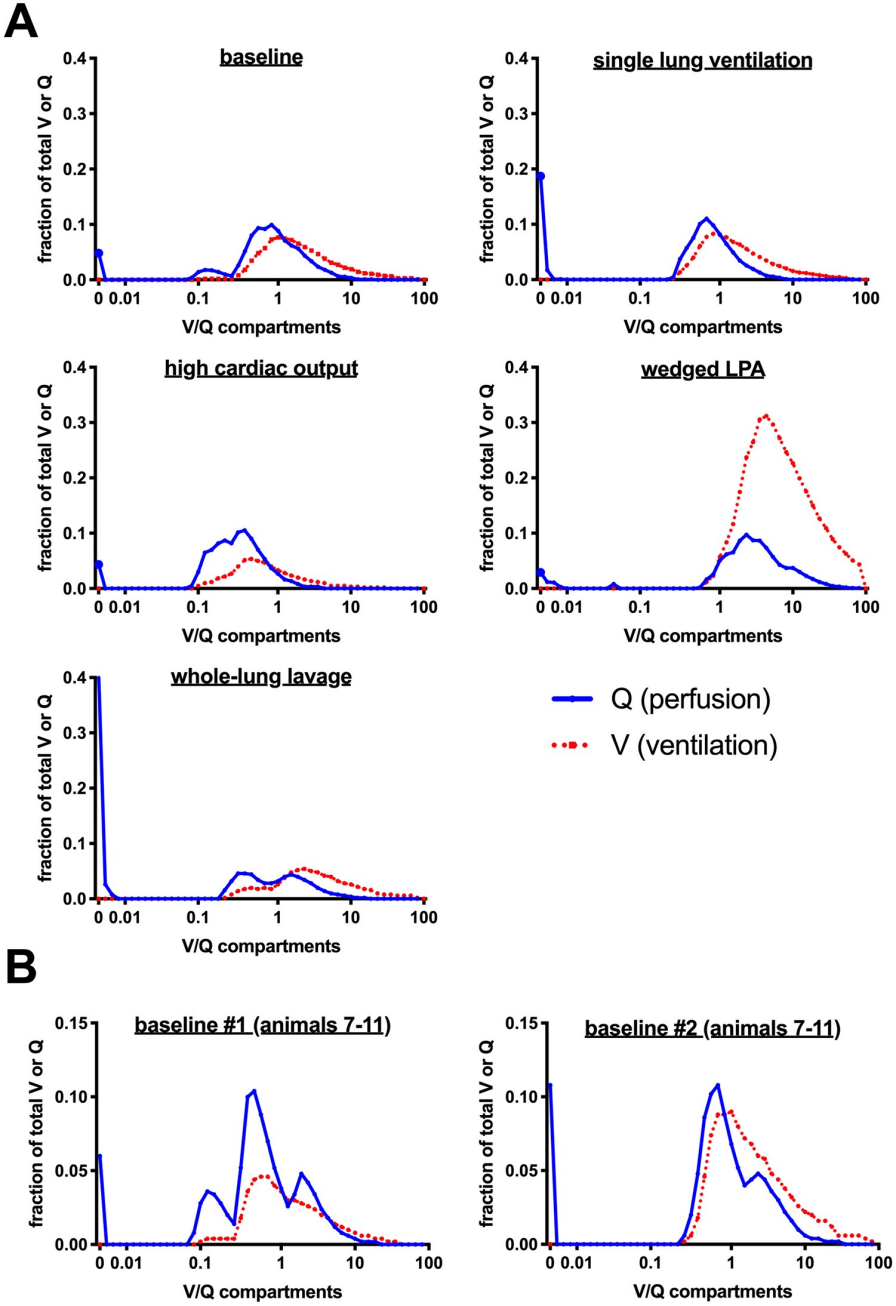
V/Q distributions measured by MMIMS-MIGET. *A*, Pooled V/Q distributions of all MMIMS-MIGET measurements and *B*, V/Q distributions of the first and second baseline conditions for animals 7–11. On the y-axis fractional perfusion (Q) and ventilation (V) with respect to cardiac output or minute ventilation are depicted. On the x-axis, the 50 V/Q compartments as measured by MMIMS-MIGET are displayed. Compartment “0” is intrapulmonary shunt. Fractional perfusion and ventilation are presented as blue lines and red dots, respectively.

Distributions of ventilation and perfusion were measured during baseline and all interventions except for the PEEP trial. At baseline, the majority of animals exhibited normal V/Q distributions. In two animals (#10 and #11), however, distributions were shifted to low V/Q regions at the start of the experiment. V/Q distributions during baseline #1 and #2 (only animals 7–11) were comparable. Our interventions produced the expected effects on V/Q distributions. Single-lung ventilation increased shunt without significant effects on overall V/Q. High cardiac output shifted the overall V/Q distribution to low V/Q compartments without increasing shunt. wedged left main pulmonary artery shifted V and Q to high V/Q compartments. repeated whole-lung lavage increased shunt and Q to low V/Q compartments but also shifted ventilation to high V/Q compartments. Shunt values acquired by MMIMS-MIGET underestimated Venous admixture, which has been previously reported [21]. V/Q distributions are shown in Fig 4.

## Discussion

Heat and water exchange within the respiratory tract may be impaired in the presence of acute V/Q-mismatch. To test this hypothesis, we measured respiratory enthalpy before and after the induction of an acute V/Q-mismatch, using four interventions (single-lung ventilation, high cardiac output, wedged left main PA and repeated whole-lung lavage) and confirmed the changes in V/Q distributions using MMIMS-MIGET. These interventions were chosen based on their potential clinical relevance. Single-lung ventilation produces atelectasis (comparable with airway obstruction or intubation of the right mainstem bronchus), high cardiac output may be present in sepsis, wedged left main PA mimics acute pulmonary embolism with significant dead space ventilation and repeated whole-lung lavage leads to surfactant depletion serving as an experimental model of ARDS with increased venous admixture, increased dead space ventilation and a shift of V/Q distributions [30]. The results of this study show that pulmonary heat exchange remained stable despite acute, clinically relevant mismatch of pulmonary ventilation and perfusion.

### Baseline observations

The baseline distributions of V/Q were generally broader than expected with some high and low V/Q compartments (Fig 3A and 3B). In some animals, V/Q distributions were multimodal, (Fig 3B). This might be attributed to the animals’ unphysiological body position (supine), the effects of anesthesia and muscle relaxation. Rightward distribution and increased dead space values in pigs were reported previously [22,31–34]. Irrespective of the individual baseline V/Q distribution, the effects of the four interventions were unequivocal. Pre-existing V/Q-mismatch at baseline does not limit the clinical correlations for two reasons. First, in clinical practice, especially in patients with diseased lungs, V/Q distributions may be wider at baseline before any acute shift in V/Q distribution occurs. Second, as an initial investigation, we did not expect to detect baseline V/Q mismatch by measuring respiratory enthalpy but rather hypothesized that respiratory enthalpy changes significantly following an acute shift in V/Q distributions.

### Single-lung ventilation

We expected lower respiratory enthalpy during single-lung ventilation due to an overall lower surface for gas exchange and an increase in shunt fraction. MMIMS-MIGET data showed a selective increase in shunt fraction, whereas overall V/Q distributions were stable. Therefore, inspired gases were delivered to a lung with almost physiological V/Q distributions. Hypoxic pulmonary vasoconstriction (HPV) may have diverted blood flow away from poorly ventilated or unventilated compartments and thereby restored V/Q matching. Generally speaking, thermodynamics of inspired gases may only reflect V/Q compartments exhibiting adequate ventilation (i.e., normal or high V/Q). Single-lung ventilation-induced intrapulmonary shunt and low-V/Q compartments are missed simply because inspired gases are not delivered to these compartments. Extrapulmonary shunt (i.e., venous admixture not occurring in the pulmonary circulation, e.g., right-left shunt due to atrial or ventricular septal defects, bronchial blood flow, Thebesian veins) might be better suited for analysis by means of thermodynamics. However, no respiratory enthalpy dynamics were observed even during the transitory phase before compensatory mechanisms, such as hypoxic pulmonary vasoconstriction, are fully developed (first 10min after establishing an intervention).

### High cardiac output and wedged pulmonary artery

During augmented cardiac output, an increase in respiratory enthalpy was expected as pulmonary blood flow provides the major source of heat in the lower respiratory tract [9], whereas reduced respiratory enthalpy was expected after occlusion of the left pulmonary artery. MMIMS-MIGET demonstrated increased perfusion to low-V/Q areas with high cardiac output, probably due to recruitment of capillaries in these compartments [26]. Serikov *et al*. showed that during normoventilation, the exhaled temperature difference correlates with cardiac output with a slope of 0.05–0.1°C/L/min [11]. On average, cardiac output increased by 7.5 ± 1.7 L/min during high cardiac output interventions in our experiments. The expected increase in temperature of ~ 0.4–0.8 °C might be too subtle in order to cause significant changes in respiratory enthalpy. After occluding the left pulmonary artery, residual heat exchange carried by the bronchial circulation might have reduced the effect of this intervention on respiratory enthalpy.

### Whole-lung lavage

Repeated whole-lung lavage produced increased venous admixture, increased dead space ventilation and a shift of V/Q distributions and was expected to decrease respiratory enthalpy. The intervention might have predominantly affected the most distal bronchioles and alveoli, where conditioning of inspired gases is usually completed. Furthermore, perfusion from systemic bronchial arteries might be sufficient for thermal conditioning during normoventilation.

### Limitations

Thermal challenges (e.g. hyperventilation with or without frigid air, a step decrease in inspired humidity, infusion of cold fluids into PA) were employed in the majority of published studies [1,2,4–6,10–14,35,36]. In contrast, we did not modulate inspired gases (gases were always delivered dry and at room temperature) and ventilation parameters were only adjusted within clinically relevant limits (i.e., according to etCO_2_ and S_p_O_2_ targets) in order to be able to study thermodynamic effects of acute V/Q-mismatch without confounding variables. Consequently, the thermal burden in these protocols was lower compared to published experiments and therefore, the respiratory system was able to compensate for the reduced thermodynamic efficiency induced by V/Q-mismatch.

Respiratory enthalpy was measured at the proximal end of the endotracheal tube. Possibly, expired enthalpy differences are greater in distal airways. For example, respiratory enthalpy differences between the left and right mainstem bronchus might be significant after wedging the left main pulmonary artery. Furthermore, heat measured at different gas flows reflects specific anatomic sites within the bronchial tree. High flows generally reflect the temperature from distal airways, whereas proximal airways contribute during low gas flows. We measured mean respiratory enthalpy. Therefore, changes in respiratory enthalpy of peak flows might have been averaged out.

We considered 10 minutes to be appropriate and sufficient for equilibration after establishing a new intervention. The observation that respiratory enthalpy did not change significantly does not confirm or refute this assumption. However, given that V/Q distributions were clearly shifted, the desired acute effects were successfully achieved. We cannot exclude that V/Q mismatch would have been more pronounced if interventions were allowed more time for equilibration. In addition, carry-over effects from one intervention to the next are possible. We therefore performed single-lung ventilation, high cardiac output and wedging of the pulmonary artery in random order. Repeated whole-lung lavage had to be performed last due to the irreversible nature of the experimental ARDS model. However, we were only interested in acute shifts of V/Q distributions and respiratory enthalpy.

The measured values for shunt were different between MMIMS-MIGET and the calculated values given by the shunt formula. Qs/Qt was generally lower for MMIMS-MIGET than calculated venous admixture, which has been observed previously [21]. Of note, the discrepancies were greater for interventions that are not associated with substantial shunt perfusion (baseline, high cardiac output and wedged left main pulmonary artery). These discrepancies occur for two reasons: First, pooling of units with low-V/Q and “pure” shunt may overestimate true shunt when the shunt equation (with the FiO2 set at 1.0) is used. Second, venous admixture does not discriminate between intra- and extrapulmonary shunt, whereas MMIMS-MIGET only measures intrapulmonary shunt [21,22]. Extrapulmonary shunt refers to venous admixture (right-left shunt) that occurs outside the pulmonary arterial circulation (e.g., cardiac right-left shunt through septal defects, Thebesian veins and bronchial circulation).

### Opportunities for further evaluation

Future studies should incorporate thermal challenges in order to increase the total thermal burden in addition to acute V/Q mismatching. Assessment of respiratory enthalpy in more distal airways and during peak expiratory gas flow may allow detection of effects on respiratory heat transfer.

### Clinical implications

An intimate relationship between heat exchange in the lungs, pulmonary perfusion and ventilation has been demonstrated by previous studies [1–5,9,10] and prompted us to investigate the distinct effect of acute V/Q mismatch on pulmonary heat exchange and whether continuous measurement of pulmonary heat exchange enables monitoring of changes in V/Q matching. The observation that pulmonary heat exchange remained stable despite acute, clinically relevant V/Q mismatch suggests that the mechanisms for conditioning of inspired air are very robust. In previous experiments, heat exchange was impaired only upon employing thermal stressors (e.g., breathing frigid air) and manipulations of pulmonary perfusion or ventilation simultaneously. These potent manipulations are not feasible in clinical practice as they may pose additional risk to patients. Therefore, monitoring of pulmonary heat exchange alone does not allow assessment of V/Q matching in the clinical setting.

### Conclusion

In conclusion, despite significant V/Q-mismatch, respiratory enthalpy did not change significantly during single-lung ventilation, high cardiac output, wedging of the left main pulmonary artery or repeated whole-lung lavage and PEEP trials. Our data suggest that clinically relevant V/Q mismatch does not impair respiratory heat exchange in the absence of additional thermal stressors.

## References

[1] McFadden ER, Denison DM, Waller JF, Assoufi B, Peacock A, Sopwith T, et al. Direct recordings of the temperatures in the tracheobronchial tree in normal man. J Clin Invest. 1982 Mar;69(3):700–5. doi: 10.1172/jci110498 7061708 PMC371028

[2] McFadden ER, Pichurko BM, Bowman HF, Ingenito E, Burns S, Dowling N, et al. Thermal mapping of the airways in humans. J Appl Physiol. 1985 Feb;58(2):564–70. doi: 10.1152/jappl.1985.58.2.564 3980358

[3] Mcfadden ER. Heat and Water Exchange in Human Airways. Am Rev Respir Dis. 1992 Nov;146(5_pt_2):S8–10. doi: 10.1164/ajrccm/146.5_Pt_2.S8 1443909

[4] McFadden ER, Pichurko BM. Intraairway thermal profiles during exercise and hyperventilation in normal man. J Clin Invest. 1985 Sep 1;76(3):1007–10. doi: 10.1172/JCI112052 4044825 PMC423970

[5] Gilbert IA, Fouke JM, McFadden ER. Intra-airway thermodynamics during exercise and hyperventilation in asthmatics. J Appl Physiol. 1988 May;64(5):2167–74. doi: 10.1152/jappl.1988.64.5.2167 3391915

[6] Serikov VB, Fleming NW, Talalov VA, Stawitcke FA. Effects of the ventilation pattern and pulmonary blood flow on lung heat transfer. Eur J Appl Physiol. 2004;91(2–3):314–23. doi: 10.1007/s00421-003-0966-4 14586583

[7] Ray DW, Ingenito EP, Strek M, Schumacker PT, Solway J. Longitudinal distribution of canine respiratory heat and water exchanges. J Appl Physiol. 1989. doi: 10.1152/jappl.1989.66.6.2788 2745342

[8] Solway J, Pichurko BM, Ingenito EP, McFadden ER, Fanta CH, Ingram RH, et al. Breathing pattern affects airway wall temperature during cold air hyperpnea in humans. Am Rev Respir Dis. 1985. doi: 10.1164/arrd.1985.132.4.853 4051320

[9] Serikov VB, Rumm MS, Kambara K, Bootomo MI, Osmack AR, Staub NC. Application of respiratory heat exchange for the measurement of lung water. J Appl Physiol. 1992. doi: 10.1152/jappl.1992.72.3.944 1568990

[10] Serikov VB, Fleming NW. Pulmonary and bronchial circulations: contributions to heat and water exchange in isolated lungs. J Appl Physiol. 2001;91(5):1977–85. doi: 10.1152/jappl.2001.91.5.1977 11641333

[11] Serikov VB, Jerome EH, Fleming NW, Moore PG, Stawitcke FA, Staub NC. Airway thermal volume in humans and its relation to body size. J Appl Physiol. 1997;83(2):668–76. doi: 10.1152/jappl.1997.83.2.668 9262466

[12] Baile EM, Dahlby RW, Wiggs BR, Paré PD, Pare PD. Role of tracheal and bronchial circulation in respiratory heat exchange. J Appl Physiol. 1985 Jan;58(1):217–22. doi: 10.1152/jappl.1985.58.1.217 3917992

[13] Solway J, Leff AR, Dreshaj I, Munoz NM, Ingenito EP, Michaels D, et al. Circulatory heat sources for canine respiratory heat exchange. J Clin Invest. 1986 Oct 1;78(4):1015–9. doi: 10.1172/JCI112655 3760181 PMC423747

[14] Gilbert IA, Regnard J, Lenner KA, Nelson JA, McFadden ER. Intrathoracic airstream temperatures during acute expansions of thoracic blood volume. Clin Sci (Lond). 1991 Nov;81(5):655–61. doi: 10.1042/cs0810655 1661652

[15] McFadden ERJ. Respiratory heat and water exchange: physiological and clinical implications. J Appl Physiol. 1983 Feb;54(2):331–6. doi: 10.1152/jappl.1983.54.2.331 6833029

[16] Wagner PD. The multiple inert gas elimination technique (MIGET). Intensive Care Med. 2008;34(6):994–1001. doi: 10.1007/s00134-008-1108-6 18421437

[17] Baumgardner JE, Choi IC, Vonk-Noordegraaf A, Frasch HF, Neufeld GR, Marshall BE. Sequential V̇A/Q̇ distributions in the normal rabbit by micropore membrane inlet mass spectrometry. J Appl Physiol. 2000. doi: 10.1152/jappl.2000.89.5.1699 11053316

[18] Dünges B, Karmrodt J, Baumgardner JE, Markstaller K. Ventilations-Perfusions-Verteilungen in der Lunge. Anaesthesist. 2007 Jun;56(6):612–6. doi: 10.1007/s00101-007-1190-0 17492417

[19] Kretzschmar M, Schilling T, Vogt A, Rothen HU, Borges JB, Hachenberg T, et al. Multiple inert gas elimination technique by micropore membrane inlet mass spectrometry—a comparison with reference gas chromatography. J Appl Physiol. 2013 Oct 15;115(8):1107–18. doi: 10.1152/japplphysiol.00072.2013 23869066

[20] Baumgardner JE, Choi IC, Vonk-Noordegraaf A, Frasch HF, Neufeld GR, Marshall BE. Sequential V(A)/Q distributions in the normal rabbit by micropore membrane inlet mass spectrometry. J Appl Physiol. 2000 Nov;89(5):1699–708. doi: 10.1152/jappl.2000.89.5.1699 11053316

[21] Duenges B, Vogt A, Bodenstein M, Wang H, Böhme S, Röhrig B, et al. A comparison of micropore membrane inlet mass spectrometry-derived pulmonary shunt measurement with riley shunt in a porcine model. Anesth Analg. 2009 Dec;109(6):1831–5. doi: 10.1213/ANE.0b013e3181bbc401 19923510

[22] Gerber D, Vasireddy R, Varadarajan B, Hartwich V, Schär MY, Eberle B, et al. Near-real-time pulmonary shunt and dead space measurement with micropore membrane inlet mass spectrometry in pigs with induced pulmonary embolism or acute lung failure. J Clin Monit Comput. 2019. doi: 10.1007/s10877-018-00245-0 30603824

[23] Lumb AB, Preceded by: Lumb AB. Nunn’s applied respiratory physiology [Internet]. 2017. 544 p.

[24] West JB (John B. Respiratory physiology: the essentials [Internet]. Wolters Kluwer Health/Lippincott Williams & Wilkins; 2012. 200 p.

[25] Schwarzkopf K, Schreiber T, Preussler NP, Gaser E, Hüter L, Bauer R, et al. Lung perfusion, shunt fraction, and oxygenation during one-lung ventilation in pigs: The effects of desflurane, isoflurane, and propofol. J Cardiothorac Vasc Anesth. 2003;17(1):73–5. doi: 10.1053/jcan.2003.13 12635064

[26] Bryan TL, Van Diepen S, Bhutani M, Shanks M, Welsh RC, Stickland MK. The effects of dobutamine and dopamine on intrapulmonary shunt and gas exchange in healthy humans. J Appl Physiol. 2012 Aug 15;113(4):541–8. doi: 10.1152/japplphysiol.00404.2012 22700799 PMC3424060

[27] Stickland MK, Tedjasaputra V, Seaman C, Fuhr DP, Collins SÉ, Wagner H, et al. Intra-pulmonary arteriovenous anastomoses and pulmonary gas exchange: Evaluation by microspheres, contrast echocardiography and inert gas elimination HHS Public Access. J Physiol. 2019;597(22):5365–84. doi: 10.1113/JP277695 31429918 PMC6858494

[28] Amundsen T, Kværness J, Aadahl P, Waage A, Bjermer L, Ødegård A, et al. A closed-chest pulmonary artery occlusion/reperfusion model in the pig: Detection of experimental pulmonary embolism with MR angiography and perfusion MR imaging. Invest Radiol. 2000. doi: 10.1097/00004424-200005000-00003 10803670

[29] Muellenbach RM, Kredel M, Bernd Z, Johannes A, Kuestermann J, Schuster F, et al. Acute respiratory distress induced by repeated saline lavage provides stable experimental conditions for 24 hours in pigs. Exp Lung Res. 2009 Apr;35(3):222–33. doi: 10.1080/01902140802534975 19337905

[30] Russ M, Kronfeldt S, Boemke W, Busch T, Francis RCE, Pickerodt PA. Lavage-induced Surfactant Depletion in Pigs As a Model of the Acute Respiratory Distress Syndrome (ARDS). J Vis Exp. 2016 Sep 7;(115). 10.3791/53610 PMC5091986 27684585

[31] Tusman G, Suarez-Sipmann F, Borges JB, Hedenstierna G, Bohm SH. Validation of bohr dead space measured by volumetric capnography. In: Applied Physiology in Intensive Care Medicine 1: Physiological Notes—Technical Notes—Seminal Studies in Intensive Care, Third Edition. 2012.

[32] Tusman G, Sipmann FS, Bohm SH. Rationale of dead space measurement by volumetric capnography. Anesth Analg. 2012. doi: 10.1213/ANE.0b013e318247f6cc 22383673

[33] Batchinsky AI, Weiss WB, Jordan BS, Dick EJ, Cancelada DA, Cancio LC. Ventilation-perfusion relationships following experimental pulmonary contusion. J Appl Physiol. 2007. doi: 10.1152/japplphysiol.00563.2006 17569766

[34] Kretzschmar M, Kozian A, Baumgardner JE, Schreiber J, Hedenstierna G, Larsson A, et al. Bronchoconstriction induced by inhaled methacholine delays desflurane uptake and elimination in a piglet model. Respir Physiol Neurobiol. 2016. 10.1016/j.resp.2015.09.014 26440992

[35] Ingenito EP, Solway J, McFadden ER, Pichurko B, Bowman HF, Michaels D, et al. Indirect assessment of mucosal surface temperatures in the airways: theory and tests. J Appl Physiol. 1987 Nov;63(5):2075–83. doi: 10.1152/jappl.1987.63.5.2075 3693240

[36] Gilbert IA, Fouke JM, McFadden ER. The effect of repetitive exercise on airway temperatures. Am Rev Respir Dis. 1990 Oct;142(4):826–31. doi: 10.1164/ajrccm/142.4.826 2221589

